# Functional Substitution of a Eukaryotic Glycyl-tRNA Synthetase with an Evolutionarily Unrelated Bacterial Cognate Enzyme

**DOI:** 10.1371/journal.pone.0094659

**Published:** 2014-04-17

**Authors:** Chin-I Chien, Yu-Wei Chen, Yi-Hua Wu, Chih-Yao Chang, Tzu-Ling Wang, Chien-Chia Wang

**Affiliations:** 1 Department of Life Sciences, National Central University, Jung-li, Taiwan; 2 Department of Neurology, Landseed Hospital, Ping-jen, Taiwan; 3 Graduate Institute of Mathematics and Science Education, National Hsinchu University of Education, Hsinchu, Taiwan; Institute of Enzymology of the Hungarian Academy of Science, Hungary

## Abstract

Two oligomeric types of glycyl-tRNA synthetase (GlyRS) are found in nature: a α_2_ type and a α_2_β_2_ type. The former has been identified in all three kingdoms of life and often pairs with tRNA^Gly^ that carries an A73 discriminator base, while the latter is found only in bacteria and chloroplasts and is almost always coupled with tRNA^Gly^ that contains U73. In the yeast *Saccharomyces cerevisiae*, a single GlyRS gene, *GRS1*, provides both the cytoplasmic and mitochondrial functions, and tRNA^Gly^ isoacceptors in both compartments possess A73. We showed herein that *Homo sapiens* and *Arabidopsis thaliana* cytoplasmic GlyRSs (both α_2_-type enzymes) can rescue both the cytoplasmic and mitochondrial defects of a yeast *grs1* ^-^ strain, while *Escherichia coli* GlyRS (a α_2_β_2_-type enzyme) and *A*. *thaliana* organellar GlyRS (a (αβ)_2_-type enzyme) failed to rescue either defect of the yeast mull allele. However, a head-to-tail αβ fusion of *E. coli* GlyRS effectively supported the mitochondrial function. Our study suggests that a α_2_-type eukaryotic GlyRS may be functionally substituted with a α_2_β_2_-type bacterial cognate enzyme despite their remote evolutionary relationships.

## Introduction

Aminoacyl-tRNA synthetases (aaRSs) establish the genetic code by coupling amino acids specifically to their cognate tRNAs. The resultant aminoacyl-tRNA is then delivered by an elongation factor to ribosomes for protein synthesis. The anticodon of a tRNA reads the codon of mRNA by forming Watson-Crick base pairing. Prokaryotes contain a basic set of 18–20 aaRs, but eukaryotes contain more, as they possess a second compartment (mitochondria) capable of protein translation [1]–[3]. For example, yeast possesses two distinct sets of aaRSs, one localized to the cytoplasm and the other to mitochondria. Each set is specific for cognate tRNAs within its respective cellular compartment, and it is sequestered from isoacceptors that are confined in other compartments. Cytoplasmic and mitochondrial isoforms of an aaRS with a given amino acid specificity are most often encoded by two distinct nuclear genes, regardless of the cellular compartments in which they are localized. However, four *Saccharomyces cerevisiae* genes, *ALA1* (which encodes alanyl-tRNA synthetase) [4], *GRS1* (which encodes glycyl-tRNA synthetase [GlyRS]) [5], *HTS1* (which encodes histidyl-tRNA synthetase) [6], and *VAS1* (which encodes valyl-tRNA synthetase) [7], specify both the cytoplasmic and mitochondrial functions. This dual-functional feature has been conserved in homologues of these genes in almost all yeast species studied [8]–[10]. In addition, the yeast *GUS1* gene also specifies both the cytoplasmic and mitochondrial functions; its protein product acts as a glutamyl-tRNA synthetase in the cytoplasm and as a non-discriminating glutamyl-tRNA synthetase in mitochondria [11].

On the basis of conserved sequence motifs, the quaternary structure, and aminoacylation function, aaRSs can be divided into two classes of 10 enzymes each [12], [13]. Class I enzymes possess two signature sequences, HIGH and KMSKS, while class II enzymes contain three degenerate motifs―motifs 1, 2, and 3. In addition, class I enzymes first couple amino acids to the 2′-OH of the terminal adenylate residue of tRNA before transferring it to the 3′-OH, while class II enzymes directly couple it to the 3′-OH. Normally, orthologous enzymes that couple the same amino acid to isoaccepting tRNAs share convincing sequence similarities in their catalytic core domains and are grouped into the same class (I or II), suggesting that they descend from a common ancestor. However, three exceptions to this rule exist: lysyl-tRNA synthetase, GlyRS, and phenylalanyl-tRNA synthetase. In the case of lysyl-tRNA synthetase, both class I- and class II-type enzymes were found [14], while in the case of GlyRS, two oligomeric forms were identified: a α_2_β_2_ heterotetramer and a α_2_ homodimer [15], [16]. Despite the fact that both forms of GlyRS possess a class II-defining architecture, they drastically differ in size and sequence [17]. As a result, they are believed to have different origins [18]. To date, the α_2_β_2_-type enzymes are found only in bacteria and chloroplasts, while the α_2_-type enzymes have been recovered from all three domains of life. In the case of phenylalanyl-tRNA synthetase, neither oligomeric structure nor modular organization is conserved over phylogeny [19].

Results obtained from study of *Escherichia coli*, *S. cerevisiae*, and *Thermus thermophilus* tRNAs^Gly^ suggest that the major identity elements of tRNA^Gly^ reside in the discriminator base (N73), the first three base pairs of the acceptor stem (1∶72, 2∶71, and 3∶70), and C35 and C36 in the anticodon loop [3]. These identity elements essentially dictate the efficiency of tRNA^Gly^ aminoacylation by its cognate enzyme [20]. The most striking difference between bacterial and eukaryotic tRNA^Gly^ isoacceptors is the discriminator base, which is nearly always a U in bacteria and an A in eukaryotic cytoplasm. For example, in *E. coli*, N73 is a U, while in *Arabidopsis thaliana*, N73 is a U in the mitochondrial-encoded tRNA^Gly^ and an A in the nuclear-encoded cytosolic tRNAs^Gly^ [21]. Typically, a α_2_β_2_-type enzyme is coupled with U73-containing tRNA^Gly^, while a α_2_-type enzyme pairs with A73-containing tRNA^Gly^ [15]. However, this once-strong correlation between the oligomeric structure of GlyRS and the type of the discriminator base is compromised by the discovery that the glycine enzyme of the bacterium *T. thermophilus* possesses a α_2_ structure yet pairs with U73-containing tRNAs^Gly^ [22].

The yeast *S. cerevisiae* possesses two distinct nuclear GlyRS genes, *GRS1* and *GRS2*. The former encodes both cytoplasmic and mitochondrial forms of GlyRS through alternative initiation of translation [5], while the latter is virtually silent and is dispensable for cell survival under normal conditions [23], [24]. As it turned out, *GRS2* is actually a stress-inducible gene, and its protein product retains a substantial level of aminoacylation activity *in vitro* [25]. Since all yeast tRNA^Gly^ isoacceptors carry the same discriminator base―A73―it is not surprising to find that a single gene, *GRS1*, can support functions in both compartments. Notably, the first three base pairs of the acceptor stem are completely different between the cytoplasmic and mitochondrial tRNA^Gly^ isoacceptors. To explore how important the discriminator base of yeast tRNAs^Gly^ really is for recognition by a α_2_-type glycine enzyme and whether an evolutionarily unrelated α_2_β_2_-type glycine enzyme can functionally substitute for the α_2_-type yeast enzyme *in vivo*, several bacterial and eukaryotic GlyRS genes were cloned, and their abilities to rescue a yeast *GRS1* knockout strain were investigated.

## Results

### Two distinct oligomeric forms of GlyRS

**Figure 1:**
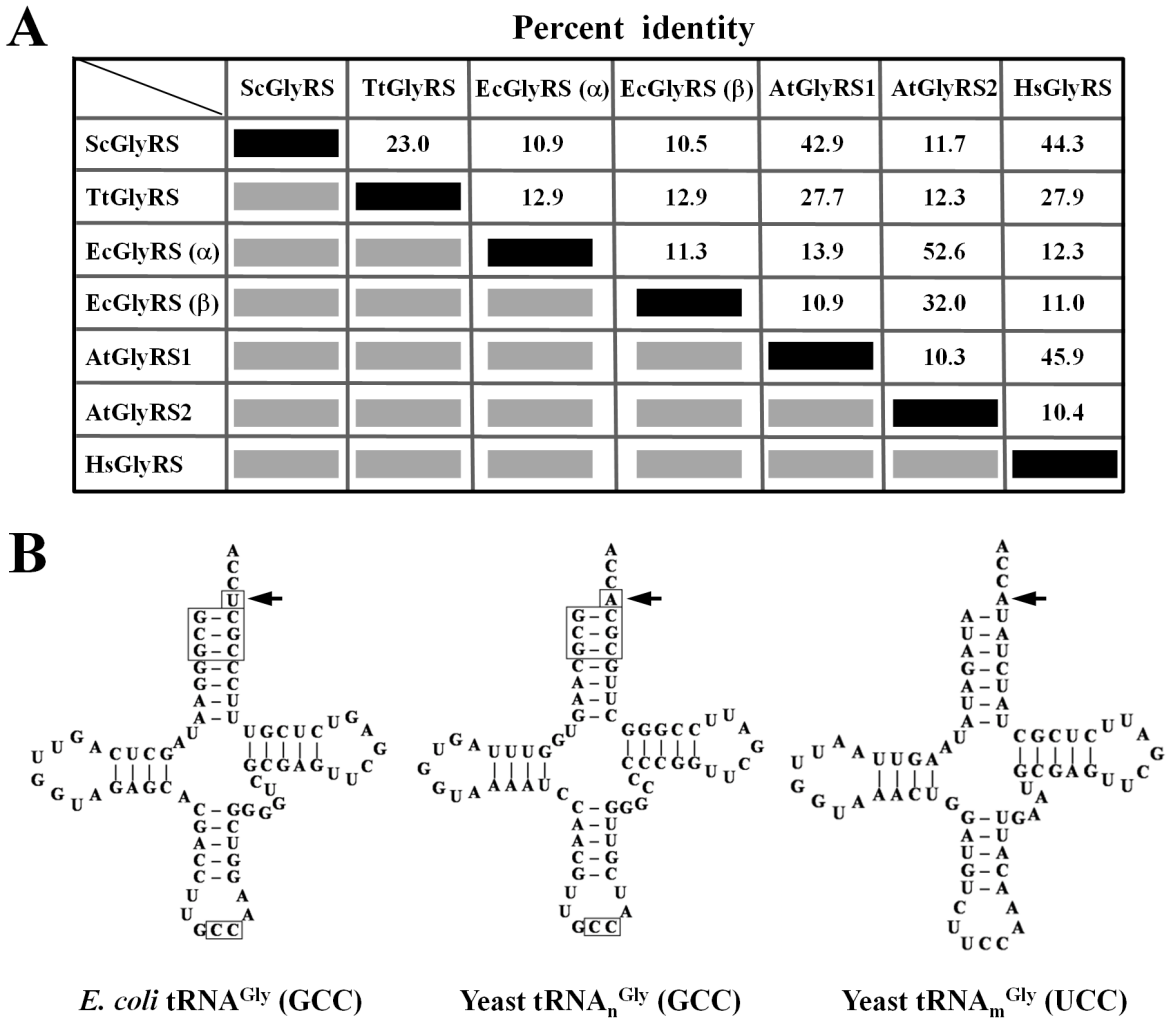
GlyRSs and tRNAs^Gly^. (**A**) Sequence homology among various GlyRSs. (**B**) Identity elements of tRNA^Gly^. Nucleotides and base pairs that were shown to be important for aminoacylation by GlyRS are boxed in *E. coli* and yeast cytoplasmic tRNAs^Gly^. The discriminator base N73 of tRNA^Gly^ is marked with an arrow. Yeast cytoplasmic and mitochondrial tRNA^Gly^ isoacceptors both possess the eukaryote-specific discriminator base A73, while *E. coli* tRNA^Gly^ contains the bacterium-specific discriminator base U73.

Two distinct oligomeric forms of GlyRS are found in nature, a α_2_ form and a α_2_β_2_ form (summarized in Table 1). These two forms exhibit low homology in sequence and size. For example, α subunit of *E. coli* GlyRS (EcGlyRS) [a α_2_β_2_-type enzyme] shares significant homology (∼53% identity) with α moiety of *A. thaliana* GlyRS2 (AtGlyRS2) [a (αβ)_2_-type enzyme], but is highly diverged (<15% identity) from α subunit of *S. cerevisiae* or *Homo sapiens* GlyRS (ScGlyRS or HsGlyRS) [both α_2_-type enzymes] (Fig. 1A). Conversely, *T. thermophilus* GlyRS (TtGlyRS) [a α_2_-type enzyme] is more closely related to α subunit of eukaryotic GlyRSs (α_2_-type enzymes) [>20% identity] than to α or β subunit of *E. coli* GlyRS or *A. thaliana* GlyRS2 (<15% identity) (Fig. 1A). In addition, the number of amino acid residues in each subunit of the representative enzymes differs drastically, ranging from 303 to 739 (Figure S1). In a α_2_-type enzyme, the class II-defining signature motifs are located in the N-terminal moiety, while the anticodon-binding domain (ABD) is located in the C-terminal moiety. In a α_2_β_2_ or (αβ)_2_-type enzyme, the class II-defining signature motifs reside in the N-terminus of α subunit, while the ABD resides in the C-terminus of β subunit (Figure S1).

**Table 1:** Oligomeric stryctures of glycyl-tRNA synthetase and the discriminator base N73 of tRNAs^Gly^.

Origin of Glycyl-tRNA synthetase	Oligomeric structure	The discriminator base N73 of tRNA^Gly^
Bacterium		
*Escherichia coli*	α_2_β_2_	U
*Synechocystis PCC6803*	α_2_β_2_	U
*Bacillus subtilis*	α_2_β_2_	U
*Lactobacillus brevis*	α_2_β_2_	U
*Bacillus thuringiensis*	α_2_	U
*Coxiella burnetii*	α_2_β_2_	U
*Chlamydia trachomatis*	(αβ)_2_	U
*Heamophilus influenzae*	α_2_β_2_	U
*Mycoplasma genitalium*	α_2_	U
*Mycoplasma pneumonia*	α_2_	U
*Mycobacterium tuberulosis*	α_2_	U
*Staphylococcus aureus*	α_2_	U
*Helicobacter pylori*	α_2_	U
*Deinococcus radiodurans*	α_2_	U
*Thermus thermophilus*	α_2_	U

Eukaryote		cytoplasm	mitochondrion	plastid
*Saccharomyces cerevisiae*	α_2_	A	A	
*Schizosaccharomyces pombe*	α_2_	A	A	
*Caenorhabditis elegans*	α_2_	A	A	
*Drosophila melanogaster*	α_2_	A	A	
*Bombyx mori*	α_2_	A	A	
*Danio rerio*	α_2_	A	A	
*Xeuopus laevis*	α_2_	A	A	
*Mus musculus*	α_2_	A	A	
*Homo sapiens*	α_2_	A	A	
*Arabidopsis thaliana*	α_2,_ (αβ)_2_	A	U	U, C

Archaebacteria				
*Aeropyrum pernix*	α_2_		A	
*Methanococcus jannaschii*	α_2_		A	
*Thermococcus kodakaraensis*	α_2_		A	

Many eukaryotes, such as *S. cerevisiae*, *Drosophila melanogaster*, *Caenorhabditis elegans*, *Bombyx mori*, and *Homo sapiens*, possess a dual-functional GlyRS gene. Two distinct protein isoforms are expected to be generated from each of these genes via alternative initiation of translation [5]. A longer form carries a mitochondrial targeting signal (MTS) at its amino-terminus and is imported into (and thus functional in) mitochondria, while a short form that lacks such a signal is confined (and thus functional) in the cytoplasm. The scenario that occurs in *A. thaliana* appears to be more sophisticated. Two distinct GlyRS enzymes are found in this plant: AtGlyRS1, which possesses a α_2_ structure, and AtGlyRS2, which possesses a (αβ)_2_ structure (Table 1 and Figure S1) [26]. AtGlyRS1 is distributed in both the cytoplasm and mitochondria but is active only in the cytoplasm. In contrast, AtGlyRS2 is confined and functional in both mitochondria and chloroplasts. The N-terminal peptide of this protein serves not only as a MTS but also as a plastidial targeting signal. Unlike *E. coli* GlyRS, which possesses a α_2_β_2_-heterotetrameric structure and is encoded by an operon that contains two open reading frames in tandem separated by a nine-nucleotide spacer, *A. thaliana* GlyRS2 possesses a unique (αβ)_2_-dimeric structure and is encoded by a single nuclear gene.

### The discriminator base N73 of glycine tRNAs

Identity elements of a tRNA are typically located at the acceptor stem and the anticodon loop. In the case of tRNA^Gly^, identity elements comprise the discriminator base (N73), the top three base pairs of the acceptor stem (1∶72, 2∶71, and 3∶70), and the last two nucleotides of the anticodon [C35 and C36] (Fig. 1B) [3], [20]. Even though the discriminator base N73 has been shown to be critical for recognition by GlyRS, bacterial and eukaryote cytoplasmic tRNAs^Gly^ contain different nucleotides at this position. The discriminator base is always a U in bacteria and an A in eukaryotic cytoplasm (Table 1). However, the mitochondrial tRNAs^Gly^ of many eukaryotes, such as *S. cerevisiae*, *Sch. pombe*, *C. elegans*, *D. melanogaster*, *B. mori*, and *H. sapiens*, contain A73 instead of U73 (Table 1), which may contribute to the finding that the same GlyRS enzyme can charge both cytoplasmic and mitochondrial tRNA^Gly^ isoacceptors in these organisms [5], [23]. In contrast, in *A. thaliana*, N73 is a U in the mitochondrial-encoded tRNA^Gly^ and is a U or a C in the chloroplast-encoded tRNA^Gly^ isoacceptors [21]. As a result, *A. thaliana* possesses two structurally distinct GlyRS enzymes―one responsible for the cytoplasm and the other for organelles. On the other hand, not all glycylation systems can fit in the two paradigms―α_2_/A73 and α_2_β_2_/U73. One such example is the GlyRS enzyme of *T. thermophilus*, which possesses a α_2_-type structure, yet pairs with U73-containing tRNAs^Gly^ (Table 1). Most strikingly, this bacterial enzyme can efficiently charge both A73 and U73-containing tRNAs^Gly^ *in vitro* [27].

### Rescue of a yeast *grs1* ^-^ strain by various bacterial GlyRSs

**Figure 2:**
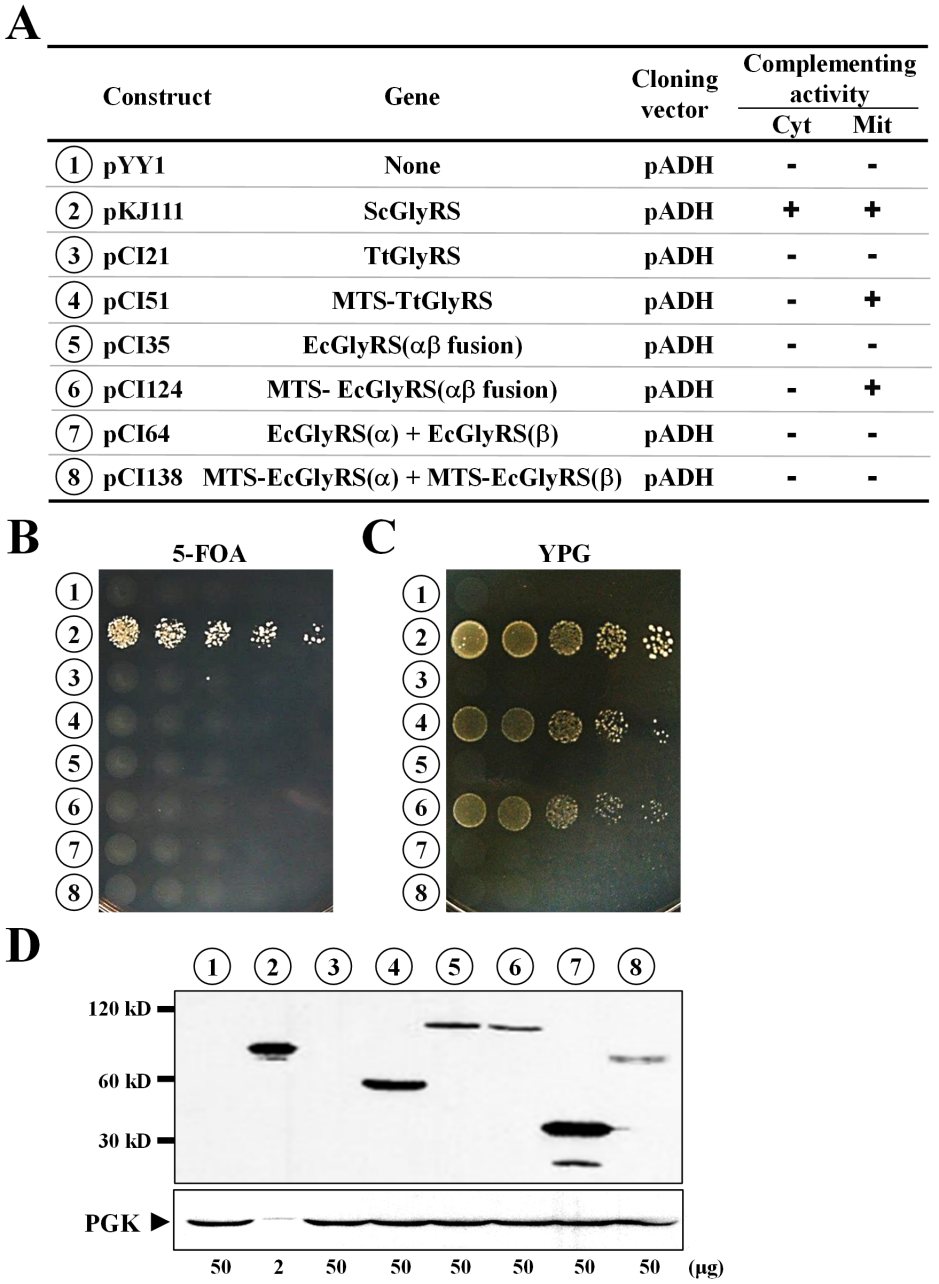
Cross-species rescue assays for bacterial GlyRSs. Constructs that carry various bacterial GlyRS genes were transformed into a *grs1* ^-^ strain of *S. cerevisiae*, and abilities of the transformants to grow on 5-FOA and YPG were tested. (**A**) Summary of the constructs and their rescue activities. The symbols “+” and “−” respectively denote positive and negative complementation. *mit*, mitochondrial; *cyt*, cytoplasmic. (**B**) Rescuing cytoplasmic activity on 5-FOA. (**C**) Rescuing mitochondrial activity on YPG. (**D**) Western blotting. The expression levels of various bacterial GlyRSs in yeast were determined via Western blotting using an anti-His_6_ tag antibody. *Upper panel*, GlyRS; *lower panel*, PGK (phosphoglycerate kinase) (as a loading control). Indicated at the bottom of the blots are the amounts of protein extracts loaded into the gel. The numbers 1∼8 (circled) in **B**∼**D** represent the constructs shown in **A**.

As *T. thermophilus* GlyRS can efficiently charge yeast tRNAs *in vitro* [27], we wondered whether this enzyme can functionally substitute for yeast *GRS1 in vivo*. As a comparison, *E. coli* GlyRS, which cannot effectively charge yeast tRNAs, was also included in the assays. To start with, the genes that encode *T. thermophilus* and *E. coli* GlyRSs were cloned in pADH or its derivative with a MTS (pWYH8). The resultant constructs were transformed into a yeast *grs1* ^-^ strain, and the abilities of the transformants to grow on 5-FOA and YPG were tested. Fig. 2 shows that TtGlyRS failed to rescue the growth defects of the knockout strain on 5-FOA and YPG, but fusion of an MTS to the bacterial enzyme enabled the fusion enzyme to restore the growth phenotype of the null allele on YPG (*numbers 3* and *4* in Fig. 2B,C). As expected, the wild-type *E. coli* GlyRS failed to substitute for either activity of yeast *GRS1 in vivo* (*numbers 7* and *8*). Unexpectedly, an engineered *E. coli* GlyRS with α and β chains fused head-to-tail into a single polypeptide, upon being imported into mitochondria, effectively rescued the growth defect of the null allele on YPG (*numbers 5* and *6*). This result positively suggests that the engineered *E. coli* enzyme can charge yeast mitochondrial tRNAs to a level that is sufficient for sustaining normal mitochondrial function.

We next examined whether the aforementioned bacterial genes were properly expressed in yeast. The constructs to be tested were transformed into a yeast strain, INVSc1, and soluble protein extracts were prepared from the resultant transformants and analyzed via Western blotting using an anti-His_6_ tag antibody. As shown in Fig. 2D, little TtGlyRS protein was detected by the Western blotting, while MTS-TtGlyRS was expressed at a level that is ∼20-fold lower than that of yeast GlyRS1 (which was also expressed under the control of an *ADH* promoter) (*numbers 3* and *4* in Fig. 2D). As expression of these two genes, TtGlyRS and MTS-TtGlyRS, was controlled by the same promoter, it appeared that this bacterial enzyme is much more stable in the mitochondria. This effect might, in turn, define its functional potential in yeast (Fig. 2C). As for expression of the engineered *E. coli* GlyRS, both EcGlyRS(αβ) and MTS-EcGlyRS(αβ) were properly expressed, with a level that is ∼25-fold lower than that of yeast GlyRS1 (*numbers 5* and *6*). The expression patterns of the wild-type *E. coli* GlyRS were somewhat more complicated. Only α subunit was detected when α and β subunits of *E. coli* GlyRS were co-expressed in the yeast cytoplasm. In contrast, only β subunit was detected when both subunits were imported into the yeast mitochondria via fusion to an MTS (*numbers 7* and *8*). Consequently, no active α_2_β_2_ complex could be formed in either compartment of the transformants, which might provide an excuse for the negative phenotype of the wild-type bacterial enzyme in the assays (Fig. 2B,C).

### Rescue of a yeast *grs1* ^-^ strain by various eukaryotic GlyRSs

**Figure 3:**
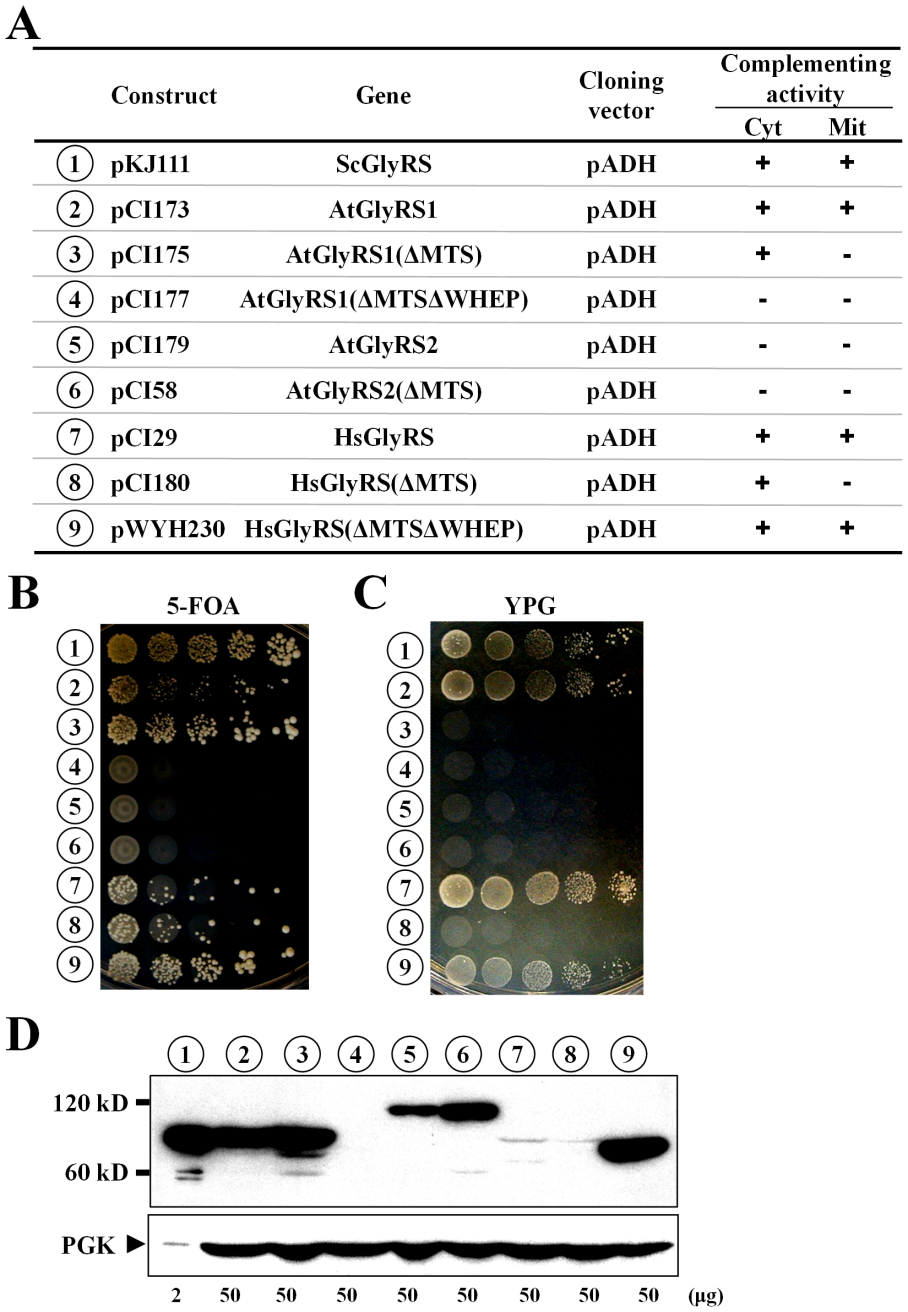
Cross-species rescue assays for eukaryotic GlyRSs. Constructs that carry various eukaryotic GlyRS genes were transformed into a *grs1* ^-^ strain of *S. cerevisiae*, and abilities of the transformants to grow on 5-FOA and YPG were tested. (**A**) Summary of the constructs and their rescue activities. The symbols “+” and “−” respectively denote positive and negative complementation. (**B**) Rescuing cytoplasmic activity on 5-FOA. (**C**) Rescuing mitochondrial activity on YPG. (**D**) Western blotting. The expression levels of various eukaryotic GlyRSs in yeast were determined via Western blotting using an anti-His_6_ tag antibody. *Upper panel*, GlyRS; *lower panel*, PGK (as a loading control). Indicated at the bottom of the blots are the amounts of protein extracts loaded into the gel. The numbers 1∼9 (circled) in **B**∼**D** represent the constructs shown in **A**.

As GlyRSs in eukaryotic cytoplasm are invariably coupled with tRNAs^Gly^ that carry an A73 discriminator base (Table 1), we next tested whether the genes that encode *H. sapiens* and *A. thaliana* GlyRSs can functionally substitute for yeast *GRS1 in vivo*. To this end, the genes that encode human GlyRS and *Arabidopsis* GlyRS1 and GlyRS2 were independently cloned in pADH, and the abilities of the resultant constructs to rescue a yeast *GRS1* knockout strain were tested on 5-FOA and YPG. As shown in Fig. 3, *A. thaliana* GlyRS1 successfully rescued the growth defects of the knockout strain on both 5-FOA and YPG, suggesting that this enzyme can charge yeast cytoplasmic and mitochondrial tRNA^Gly^ isoacceptors to a substantial level. Removal of the putative MTS (N-terminal amino acid residues 1-39) from AtGlyRS1, resulting in AtGlyRS1(ΔMTS), effectively eliminated its mitochondrial function, but it slightly enhanced its cytoplasmic activity (*number 3*). This finding is consistent with our assumption that the MTS-deleted AtGlyRS1 enzyme exclusively acts in the cytoplasm. Further removal of the WHEP domain from AtGlyRS1 completely abolished its rescue activities (*number 4*). In contrast with AtGlyRS1, the full-length or the MTS-deleted AtGlyRS2 enzyme failed to support either function in the assays (*numbers 5* and *6*).

We next tested human GlyRS for its cross-species rescue activities. The full-length human enzyme feebly supported the growth of the *grs1* ^-^ strain on 5-FOA, but it efficiently rescued the growth defect of the knockout strain on YPG (*number 7*). Removal of the MTS from HsGlyRS selectively impaired its mitochondrial activity (*number 8*). Most remarkably, removal of both the MTS and the WHEP domain from HsGlyRS led to a truncated enzyme that robustly supported the growth of the yeast knockout strain on both 5-FOA and YPG (*number 9*).

To gain further insight into the expression levels of these eukaryotic enzymes in yeast, Western blotting using an anti-His_6_ tag antibody was carried out. As shown in Fig. 3D, AtGlyRS1 and AtGlyRS1(ΔMTS) were properly expressed in yeast, with a level that is ∼25-fold lower than that of yeast GlyRS1. However, deletion of both the MTS and the WHEP domain knocked off its expression (*number 4*). This finding provides a rational basis as to why AtGlyRS1(ΔMTSΔWHEP) failed to support either activity in the rescue assays. Similarly, both AtGlyRS2 and AtGlyRS2(ΔMTS) were properly expressed in yeast, with a level that is ∼25–50-fold lower than that of yeast GlyRS1 (*numbers 5* and *6*). Thus, the negative phenotype of these two constructs in complementation is likely to be the effect of poor cross-species glycylation rather than defective protein expression. Contrary to the scenarios of *Arabidopsis* GlyRS1 and GlyRS2, only a faint protein band was detected for the full-length and the MTS-deleted human GlyRS enzymes (*numbers 7* and *8*). Interestingly, even with such a low level of protein expression, human GlyRS could support the growth of the yeast null allele (*numbers 7* and *8* in Fig. 3C). Most strikingly, deletion of both the MTS and the WHEP domain from HsGlyRS drastically enhanced its protein expression level [∼25-fold lower than that of yeast GlyRS1] (*number 9*), which may partially account for the increased cytoplasmic activity (Fig. 3B). It is still a puzzle as to why this truncated enzyme regained a mitochondrial activity. Perhaps, a second, cryptic MTS is embedded inside its internal sequence, and such a signal can be functional only when the protein is highly expressed. Another possibility is that the second cryptic MTS is masked by the WHEP domain and exposed upon removal of the latter.

### Cellular distributions of human GlyRS in yeast

**Figure 4:**
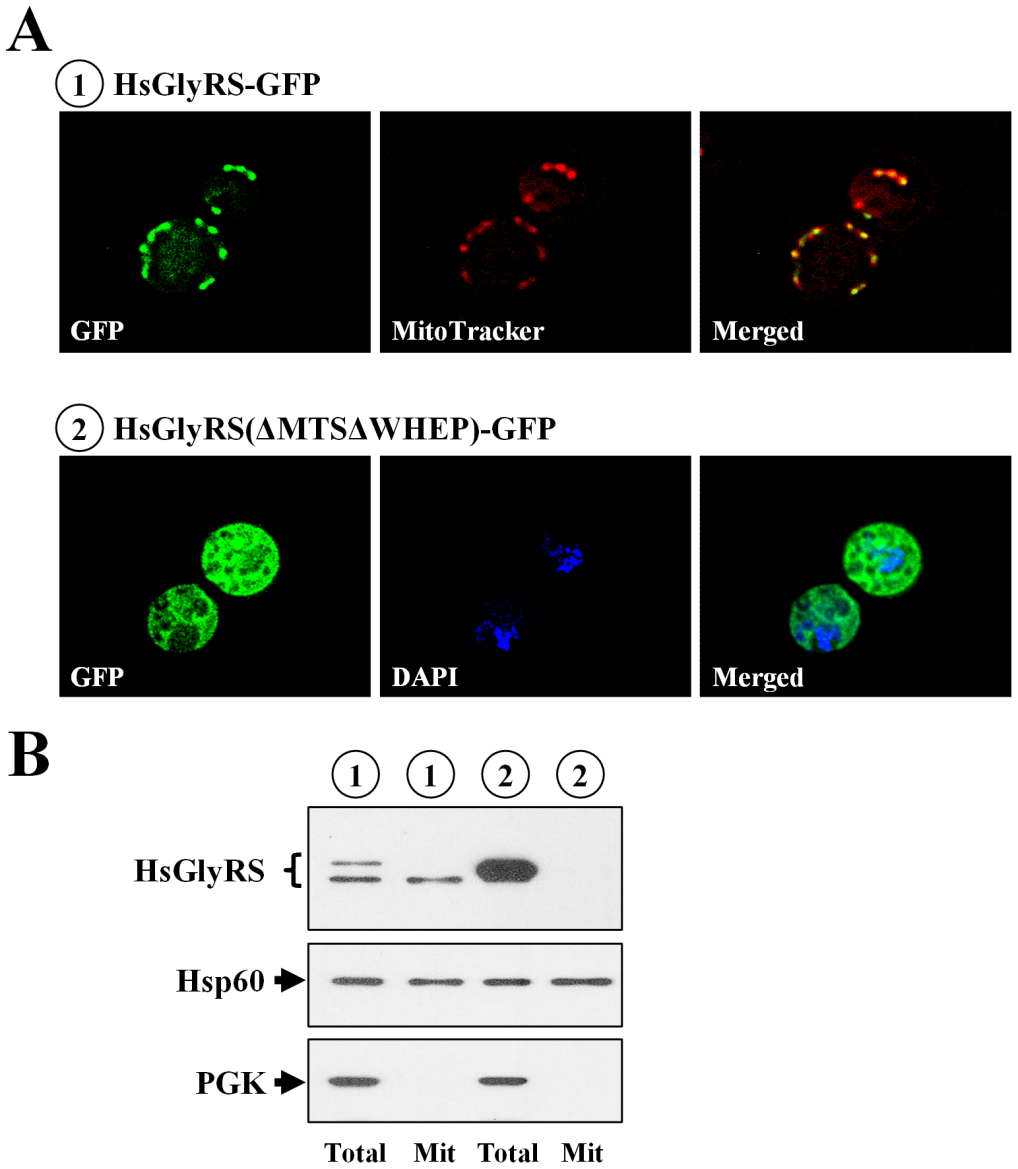
Cellular distributions of human GlyRS. Subcellular localization of the wild-type or truncated human GlyRS in yeast was determined via fluorescence microscopy or fractionation. (**A**) Fluorescence microscopy. Constructs that express HsGlyRS-GFP or HsGlyRS(ΔMTSΔWHEP)-GFP fusion proteins were first transformed into a yeast strain, INVSc1. The resultant transformants were then treated with a mitochondrion-specific dye (MitoTracker) or 4',6-diamidino-2-phenylindole (DAPI), and examined through fluorescence microscopy. MitoTracker and DAPI were respectively used to label mitochondria and nuclei. (**B**) Fractionation. Total (Total) and mitochondrial (Mit) protein extracts were prepared from the yeast transformants possessing HsGlyRS-GFP or HsGlyRS(ΔMTSΔWHEP)-GFP. The numbers 1∼2 (circled) in **B** represent the constructs shown in **A**.

The MTS used in targeting the bacterial enzymes to mitochondria was grafted from the mitochondrial precursor form of yeast valyl-tRNA synthetase, and its mitochondrial targeting potential has been repeatedly tested and confirmed [23], [28]. To evaluate whether the putative MTS of human GlyRS also efficiently acts as a targeting signal in yeast, a fluorescence microscopic analysis was carried out. A DNA sequence that encodes the green fluorescence protein (GFP) was amplified by a PCR and inserted in-frame into an appropriate site at the 3′ end of HsGlyRS and HsGlyRS(ΔMTSΔWHEP), yielding HsGlyRS-GFP and HsGlyRS(ΔMTSΔWHEP)-GFP, respectively. The resultant fusion constructs were then transformed into INVSc1, and cellular distributions of these proteins were examined via fluorescence microscopy. Expression of these GFP fusion proteins was driven by a constitutive *ADH* promoter. As shown in Fig. 4A, the full-length human GlyRS was largely confined in mitochondria (see HsGlyRS-GFP), while the truncated form of human GlyRS was predominantly localized in the cytoplasm [see HsGlyRS(ΔMTSΔWHEP)-GFP]. Due to constraints of the imaging technology used, the possibility that a minor portion of the GFP fusion proteins was targeted to other unintended cellular compartments could not be ruled out. Nevertheless, this assay explicitly demonstrates the mitochondrial targeting potential of the native MTS of human GlyRS in yeast. Thus, the higher cytoplasmic rescue activity of the WHEP-deleted human GlyRS may be the combinatorial effect of higher protein expression and preferential cytoplasmic localization of the protein. A fractionation assay further confirmed that HsGlyRS-GFP can be imported into mitochondria, but HsGlyRS(ΔMTSΔWHEP)-GFP cannot (Fig. 4B).

### Aminoacylation of yeast mitochondrial tRNA^Gly^ by *E. coli* GlyRS

**Figure 5:**
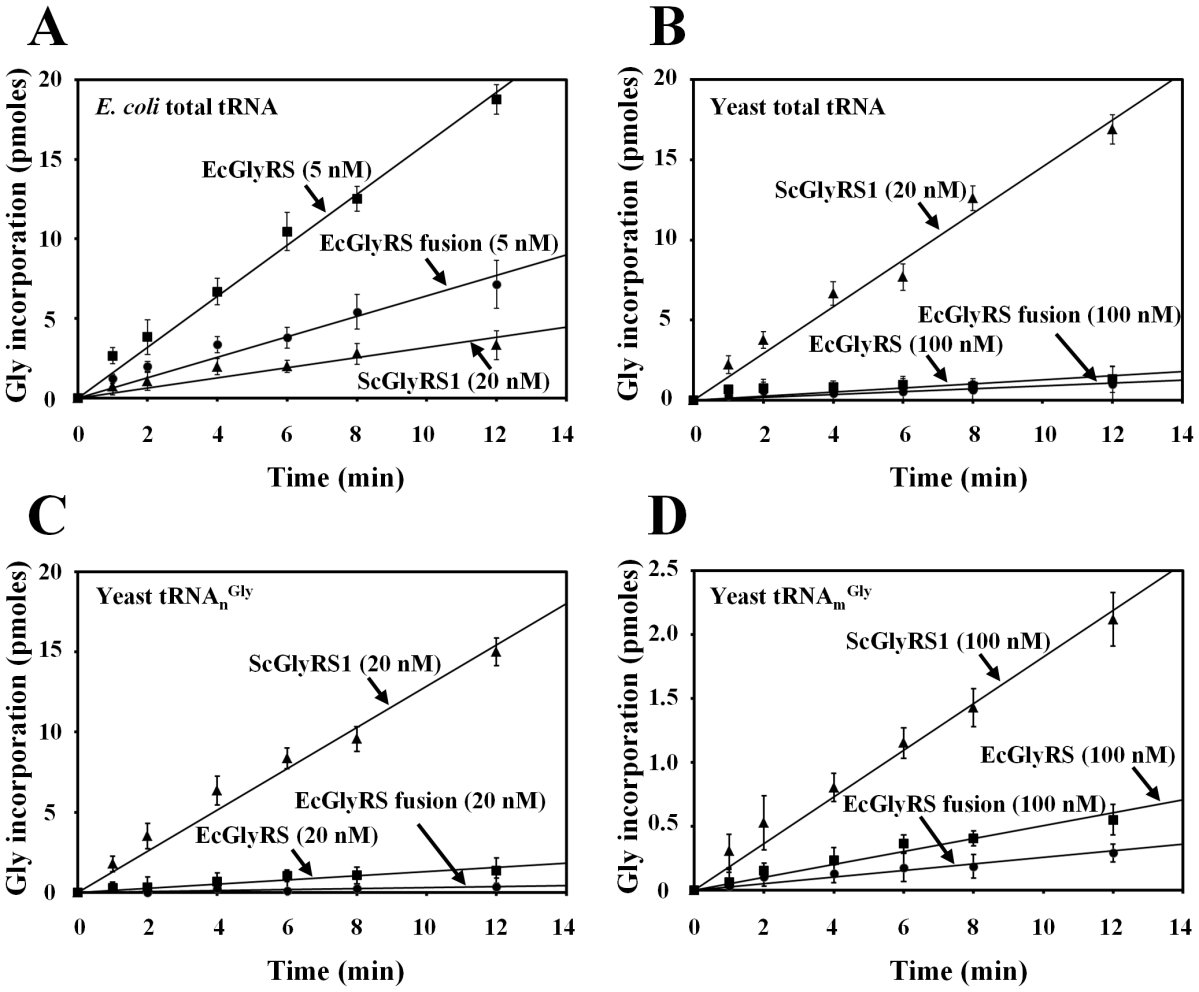
Aminoacylation activities of GlyRSs. Aminoacylation activities of purified recombinant GlyRSs were determined by measuring the relative amounts of ^3^H-glycine that were incorporated into tRNA using a liquid scintillation counter. (**A**) Glycylation of unfractionated *E. coli* tRNAs. (**B**) Glycylation of unfractionated yeast tRNAs. (**C**) Glycylation of *in vitro*-transcribed yeast tRNA_n_^Gly^. (**D**) Glycylation of *in vitro*-transcribed yeast tRNA_m_^Gly^. The final concentration of GlyRS used in the reactions was 5, 20, or 100 nM as indicated in the parenthesis. Data were obtained from three independent experiments and averaged.

To compare the relative aminoacylation activities of the wild-type and the fusion *E. coli* GlyRSs, recombinant His_6_-tagged GlyRS enzymes were purified from *E. coli* transformants via Ni-NTA column chromatography. As a reference, yeast GlyRS1 was also purified and tested. As shown in Fig. 5A, the engineered bacterial enzyme efficiently charged its cognate tRNAs, with efficiency ∼2-fold lower than that of its wild-type counterpart. In contrast, yeast GlyRS1 only poorly charged *E. coli* tRNAs. To check whether the bacterial fusion enzyme is more active than its wild-type counterpart in glycylating yeast mitochondrial tRNA^Gly^, yeast tRNA_m_^Gly^ (mitochondrial-encoded mitochondrial tRNA^Gly^) was prepared by *in vitro* transcription. As a control, yeast tRNA_n_^Gly^ (nuclear-encoded cytoplasmic tRNA^Gly^) was also *in vitro*-transcribed and tested. As shown in Fig. 5, yeast GlyRS1, which efficiently glycylated unfractionated yeast tRNAs (Fig. 5B) and *in vitro*-transcribed yeast tRNA_n_^Gly^ (Fig. 5C), feebly charged *in vitro*-transcribed yeast tRNA_m_^Gly^ (Fig. 5D). Evidently, tRNA_m_^Gly^ was a relatively poor substrate for glycylation by yeast GlyRS1, with efficiency at least 30-fold lower than that of tRNA_n_^Gly^ under the conditions used. As a result, only an almost negligible glycylation activity toward tRNA_m_^Gly^ was detected for the wild-type and the fusion *E. coli* enzymes (Fig. 5D). Hence, the fusion approach *per se* did not confer the bacterial enzyme a higher glycylation activity toward yeast tRNA_m_^Gly^. On the contrary, the observed rescue activity may merely reflect the ability of the fusion polypeptide to form a stable and active (αβ)_2_ complex in yeast mitochondria (Fig. 2), and such a low activity toward yeast tRNA_m_^Gly^ might be sufficient for sustaining normal mitochondrial function.

## Discussion

AaRSs belong to an ancient group of essential translation enzymes that establish the genetic code by coupling amino acids specifically to their cognate tRNAs. However, contrary to long-standing beliefs that a housekeeping gene is highly and constitutively expressed to satisfy the need of viable cell growth, a fairly low level of aminoacylation activity (30 to 50-fold lower than normal) is sufficient for conferring a near wild-type growth phenotype to a yeast strain defective in a specific aaRS activity. Examples of this type include yeast *ALA1* [29], *GLN4* [28], [30], *GRS1* [23], [25], and *VAS1* genes [31]. Such a feature may also contribute to the feasibility of cross-species complementation reported herein (Figs. 2 and 3). Despite the fact that *T. thermophilus* GlyRS can efficiently charge yeast cytoplasmic tRNA^Gly^ *in vitro* [27], it failed to provide the cytoplasmic function in yeast, presumably due to misfolding or instability of the protein in the yeast cytoplasm (Fig. 2). Nevertheless, fusion of an MTS to this bacterial enzyme not only enabled the fusion protein to be imported into mitochondria but also enhanced its steady-state protein level. As a result, the MTS fusion enzyme successfully conferred a respiratory phenotype to a yeast strain that was defective in mitochondrial GlyRS activity (Fig. 2). Thus, a protein that is stable in one cellular compartment may be unstable in another, which, in turn, defines its functional potential *in vivo*.

It was previously shown that fusion of α and β chains of *E. coli* GlyRS gives rise to a functional enzyme, with a substantial level of aminoacylation activity [32]. Consistent with this observation, the fusion enzyme reported herein retains an aminoacylation activity ∼2-fold lower than that of its wild-type counterpart (Fig. 5). This result underscores the possibility that the fusion enzyme adopts an active conformation, presumably a (αβ)_2_ structure. In contrast with previous observations that a α_2_β_2_-type GlyRS cannot recognize tRNAs^Gly^ with an A73 discriminator base, the bacterial fusion enzyme reported herein effectively rescued the mitochondrial defect of a yeast *GRS1* knockout strain (Fig. 2), suggesting that it can glycylate the yeast mitochondrial tRNA^Gly^ to a level that is sufficient for maintaining normal mitochondrial function. Paradoxically, the fusion enzyme does not possess a glycylation activity toward yeast mitochondrial tRNA^Gly^ higher than that of its wild-type counterpart *in vitro* (Fig. 5). Moreover, the fusion enzyme failed to rescue the cytoplasmic defect of the null allele (Fig. 2) despite the fact that yeast cytoplasmic tRNAs^Gly^ share more identity elements with *E. coli* tRNAs^Gly^ than does their mitochondrial isoacceptor (Fig. 1B). Perhaps, a higher level of aminoacylation activity (and protein translation) is required for the cytoplasm to sustain viable cell growth.

In the yeast *S. cerevisiae*, a single GlyRS gene specifies both the cytoplasmic and mitochondrial functions [5]. A similar scenario was found in the GlyRS genes of *Sch. pombe*, *C. elegans*, *D. melanogaster*, *B. mori*, and *H. sapiens* [23]. As it happens, the discriminator base N73 of both the nuclear- and mitochondrial-encoded tRNA^Gly^ isoacceptors (tRNA_n_^Gly^ and tRNA_m_^Gly^, respectively) is an A in these eukaryotes (Table 1). Such a coincidence appears to be an outcome of co-evolution between GlyRS and its cognate tRNAs. In contrast, the scenario that occurs in *A. thaliana* is much more complicated. In *A. thaliana*, N73 is an A in the nuclear-encoded tRNAs^Gly^, a U in the mitochondrial-encoded tRNA^Gly^, and a U or a C in the chloroplast-encoded tRNAs^Gly^ [21]. Nonetheless, a nuclear-encoded tRNA^Gly^ (with A73) is imported from the cytosol into the mitochondria of this plant for function. Correspondingly, *A. thaliana* possesses two distinct GlyRS enzymes: AtGlyRS1 (a α_2_-dimeric enzyme) and AtGlyRS2 [a (αβ)_2_–dimeric enzyme] [26]. AtGlyRS1 prefers A73-containing tRNA^Gly^, while AtGlyRS2 was predicted to glycylate both the organelle-encoded tRNAs^Gly^ and the imported tRNA^Gly^ present in mitochondria [26]. Contrary to this prediction, our results show that only AtGlyRS1 can functionally substitute for yeast *GRS1 in vivo* (Fig. 3). It has yet to be determined whether AtGlyRS2 really charges the imported tRNA^Gly^ (with A73).

*GARS* is a dual-functional human GlyRS gene that specifies both the cytoplasmic and mitochondrial functions [33]. Recent studies revealed that human GlyRS is involved in pathways beyond aminoacylation. This protein specifically binds to the poliovirus internal ribosome entry site, thereby facilitating the 5′ end-independent internal initiation of translation [34]. In addition, human GlyRS can be secreted from macrophages in response to Fas ligand released from tumor cells, and in turn participate in defense against ERK-activated tumor formation [35]. Moreover, specific mutations in *GARS* lead to a neurodegenerative disorder, Charcot-Marie-Tooth disease type 2D (CMT2D) [36]. Our study suggests that yeast may serve as a platform for functional analysis of the human *GARS* gene. Despite the fact that the full-length human GlyRS was poorly expressed in yeast, it successfully rescued both the cytoplasmic and mitochondrial defects of a yeast *GRS1* knockout strain, albeit with a weaker cytoplasmic rescue activity. This weak rescue activity could be improved via removal of its N-terminal 120 residues; the resultant enzyme [HsGlyRS(ΔMTSΔWHEP)] had a higher protein expression level and robustly rescued both defects of the yeast knockout strain (Fig. 3). Thus, the WHEP-truncated form of human GlyRS may be preferable over the full-length enzyme for characterizing the CMT2D-causing mutants in yeast. However, regardless of the detailed interpretation, our study suggests that the functional correlation between the oligomeric structure of GlyRS and the discriminator base of tRNA^Gly^ may be looser than previously anticipated [20].

## Materials and Methods

### Plasmid construction

Cloning of the *GRS1* gene of *S. cerevisiae* into pADH (a high-copy-number yeast shuttle vector with a constitutive *ADH* promoter, followed by a multiple cloning site that contains PstI-EagI-SpeI-NdeI-XhoI restriction sites and a short sequence coding for a His_6_ tag) was previously described [5], [23]. To clone the *GARS* gene of *Homo sapiens*, a DNA sequence extending from base pair +1 to +2217 relative to ATG1 of this gene was amplified by a reverse transcription-polymerase chain reaction (RT-PCR) as a NdeI-XhoI fragment using a pair of gene-specific primers, digested with the restriction enzymes NdeI and XhoI, and cloned into the NdeI/XhoI sites of pADH. Cloning of truncated versions of the human *GARS* gene followed a similar protocol. To introduce a mitochondrial targeting signal (MTS) into pADH, a DNA sequence that encodes the N-terminal amino acid residues 1-46 of the mitochondrial precursor form of yeast valyl-tRNA synthetase (base pairs +1∼+138 relative to ATG1) was amplified by a PCR as an XbaI-SpeI fragment, and it was inserted in-frame into the SpeI site of pADH. The resultant construct pWYH8 can then be used to express an MTS fusion protein under the control of an *ADH* promoter. Cloning of the genes that encode *Thermus thermophilus* GlyRS and *Arabidopsis thaliana* GlyRS1 and GlyRS2 followed a similar strategy.

To co-express α and β subunits of *E. coli* GlyRS in yeast, the open reading frames that encode these two subunits were cloned in a dual-promoter vector, pCI55. pCI55 is a high-copy-number yeast shuttle vector with a *GAP* (followed by a multiple cloning site that contains XbaI-BamHI-KpnI-SacI-SalI restriction sites and a short sequence coding for a His_6_ tag) and an *ADH* promoter (followed by a multiple cloning site that contains PstI-EagI-SpeI-NdeI-XhoI restriction sites and a short sequence coding for a His_6_ tag). The open reading frames that encode these two subunits were respectively amplified by a PCR as an SpeI-XhoI and an NdeI-XhoI fragment, and they were respectively cloned in the XbaI/SalI and NdeI/XhoI sites downstream of the *GAP* and *ADH* promoters. Insertion of an MTS into the dual-promoter vector followed a protocol described earlier. To fuse α and β chains into a single polypeptide, the open reading frames that encode these two subunits were respectively amplified by a PCR as an SpeI-NdeI and an NdeI-XhoI fragment and simultaneously cloned into pADH or pWYH8 (resulting in pCI35 and pCI124, respectively). The head-to-tail fusion gene constructed in this way encodes a long polypeptide with a tetrapeptide linker (α-Lys-Glu-Ala-His-β).

To generate the green fluorescence protein (GFP) fusion constructs HsGlyRS-GFP and HsGlyRS(ΔMTSΔWHEP)-GFP, the open reading frame that encodes GFP was amplified by a PCR as an XhoI-SalI fragment, digested with the restriction enzymes XhoI and SalI, and inserted in-frame into the XhoI site of pCI29 or pWYH230 (resulting in pCI195 and pCI196, respectively).

### Rescue assay for the cytoplasmic function of *GRS1*

The yeast *GRS1* knockout strain, RJT3/II-1, was previously described [24]. Complementation assays for cytoplasmic GlyRS activity were carried out by introducing a test plasmid that carries the gene of interest and a *LEU2* marker into RJT3/II-1, and the ability of the transformants to grow in the presence of 5-FOA was determined. Starting from a cell density of 1.0 *A*_600_, cell cultures were 5-fold serially diluted, and 10-µl aliquots of each dilution were spotted onto the designated plates containing 5-FOA. Plates were incubated at 30°C for 3∼5 days. The transformants evicted the maintenance plasmid with a *URA3* marker in the presence of 5-FOA, and thus could not grow on the selection medium unless a functional cytoplasmic GlyRS was encoded by the test plasmid.

### Rescue assay for the mitochondrial function of *GRS1*

RJT3/II-1 was co-transformed with a test plasmid (carrying a *LEU2* marker) and a second maintenance plasmid (carrying a *TRP1* marker) that only expresses the cytoplasmic form of GlyRS (due to a mutation in the initiator codon of the mitochondrial form). In the presence of 5-FOA, the first maintenance plasmid (carrying a *URA3* marker) was evicted from the co-transformants, while the second maintenance plasmid was retained. Thus, all co-transformants survived 5-FOA selection, due to the presence of the cytoplasmic GlyRS derived from the second maintenance plasmid. The mitochondrial phenotypes of the co-transformants were further tested on YPG plates at 30°C, with results documented on day 3 following plating. Because a yeast cell cannot survive on glycerol without functional mitochondria, the co-transformants did not grow on the YPG plates unless a functional mitochondrial GlyRS was generated from the test plasmid.

### Western blotting

The protein expression patterns of the constructs used in the complementation assays were determined via a chemiluminescence-based Western blot analysis. INVSc1 was first transformed with the constructs of interest, and total protein extracts were prepared from the transformants. Aliquots of the protein extracts (2 or 50 µg) were loaded onto a mini gel (size: 8×10 cm) that contains 10% polyacrylamide and electrophoresed at 100 V for ∼1 h. Following electrophoresis, the resolved proteins were transferred using a semi-dry transfer device to a PVDF membrane in a buffer containing 30 mM glycine, 48 mM Tris base (pH 8.3), 0.037% sodium dodecylsulfate, and 20% methanol. The membrane was probed with an HRP-conjugated anti-His_6_ tag antibody, and then exposed to x-ray film following the addition of appropriate substrates.

### Fluorescence microscopy

Fluorescence microscopy followed a protocol described earlier [28]. Yeast transformants were grown to OD_600_∼0.6 in SD/-Leu selective medium. Cells were pretreated with MitoTracker (∼300 nM) or 4',6-diamidino-2-phenylindole (DAPI) (∼0.9 nM) for 30 min. The samples were then examined through fluorescence microscopy (Axio observer.A1; Carl Zeiss, Inc.) using a 100x objective at 25°C and images were captured with a CCD camera (Axiocam MRm; Carl Zeiss, Inc.). Individual and merged images of GFP, DAPI, and MitoTracker were generated with AxioVision Rel. 4.8 software, and then subjected to 2D deconvolution with AutoQuant X2.

### Aminoacylation assay

Aminoacylation reactions were carried out at 25°C in a buffer that contains 50 mM HEPES (pH 7.5), 50 mM KCl, 15 mM MgCl_2_, 5 mM dithiothreitol, 10 mM ATP, 0.1 mg/ml bovine serum albumin (BSA), 100 µM unfractionated yeast tRNA (Boehringer Mannheim, Germany) or 5 µM *in vitro*-transcribed yeast tRNA^Gly^, and 24 µM glycine (4 µM ^3^H-glycine; Moravek Biochemicals, Brea, CA). The specific activity of ^3^H-glycine used was 35.0 Ci/mmol. Purification of GlyRS and *in vitro* transcription of tRNA followed protocols described earlier [28], [37]. Determination of the active protein concentrations by active site titration was as previously described [38]. Reactions were quenched by spotting 10 µl aliquots of the reaction mixture onto Whatman filters (Maidstone, UK) soaked in 5% trichloroacetic acid and 1 mM glycine. Filters were washed three times for 15 min each in ice-cold 5% trichloroacetic acid prior to liquid scintillation counting. Data were obtained from three independent experiments and averaged. Error bars indicate (±2 x standard deviation).

## Supporting Information

Figure S1 **Oligomeric structures of GlyRSs.** Schematic diagrams show functional domains or sequence motifs of GlyRS, including MTS (mitochondrial targeting signal), ABD (anticodon-binding domain), WHEP (a helix-turn-helix domain originally found in eukaryotic WRS, HRS, and EPRS), and class II-defining signature motifs 1, 2, and 3. The number of amino acids (aa) in each subunit of GlyRS is indicated in the parenthesis. ScGlyRS1, *Saccharomyces cerevisiae* GlyRS1; TtGlyRS, *Thermus thermophilus* GlyRS; HsGlyRS, *Homo sapiens* GlyRS; AtGlyRS, *Arabidopsis thaliana* GlyRS; EcGlyRS, *Escherichia coli* GlyRS. MTS of ScGlyRS1: aa 1-23; MTS and WHEP of AtGlyRS1: aa 1-39 and 40-116, respectively; MTS of AtGlyRS2: aa 1-58; MTS and WHEP of HsGlyRS: aa 1-54 and 55-120, respectively.
(TIF)
