# Supplementary figures and images for: Functional Substitution of a Eukaryotic Glycyl-tRNA Synthetase with an Evolutionarily Unrelated Bacterial Cognate Enzyme
Source: PLoS One. 2014 Apr 17;9(4):e94659. doi: 10.1371/journal.pone.0094659 (PMC3990555; doi:10.1371/journal.pone.0094659)

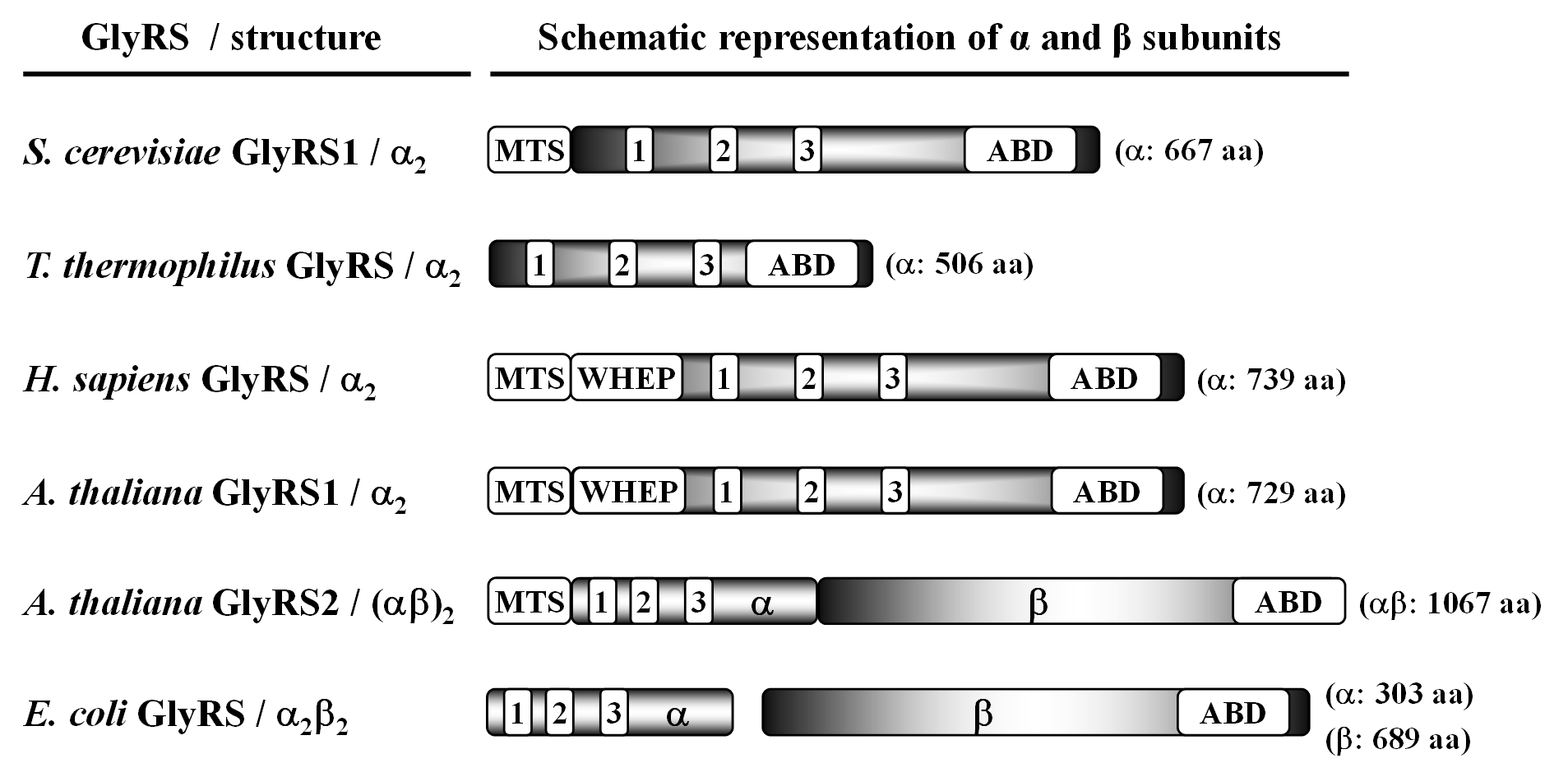

Supplement: Figure S1 — Oligomeric structures of GlyRSs. Schematic diagrams show functional domains or sequence motifs of GlyRS, including MTS (mitochondrial targeting signal), ABD (anticodon-binding domain), WHEP (a helix-turn-helix domain originally found in eukaryotic WRS, HRS, and EPRS), and class II-defining signature motifs 1, 2, and 3. The number of amino acids (aa) in each subunit of GlyRS is indicated in the parenthesis. ScGlyRS1, Saccharomyces cerevisiae GlyRS1; TtGlyRS, Thermus thermophilus GlyRS; HsGlyRS, Homo sapiens GlyRS; AtGlyRS, Arabidopsis thaliana GlyRS; EcGlyRS, Escherichia coli GlyRS. MTS of ScGlyRS1: aa 1-23; MTS and WHEP of AtGlyRS1: aa 1-39 and 40-116, respectively; MTS of AtGlyRS2: aa 1-58; MTS and WHEP of HsGlyRS: aa 1-54 and 55-120, respectively. (TIF) [file pone.0094659.s001.tif]
